# Rifampicin in Osteoarticular Infections: A Comprehensive Review of Current Evidence

**DOI:** 10.3390/antibiotics15090872

**Published:** 2026-09-07

**Authors:** France Laurent, Jean Cyr Yombi

**Affiliations:** 1 Department of Internal Medicine and Infectious Diseases, Centre Hospitalier Universitaire (CHU) Helora, 7000 Mons, Belgium; france.laurent@helora.be; 2 Department of Internal Medicine and Infectious Diseases, Cliniques Universitaires Saint-Luc, UCLouvain, 1200 Brussels, Belgium

**Keywords:** rifampicin, osteoarticular infection, prosthetic joint infection, osteomyelitis, fracture-related infection, biofilm, *Staphylococcus aureus*, antibiotic resistance

## Abstract

Background. Osteoarticular infections (OAI)—including prosthetic joint infections (PJI), fracture-related infections (FRI), and osteomyelitis—are challenging to cure because bacterial biofilm on implant surfaces confers up to 1000-fold antibiotic tolerance, driving chronicity and relapse. Rifampicin, through its bactericidal activity against quiescent, biofilm-embedded bacteria and excellent oral bioavailability, occupies a pivotal role in managing Gram-positive OAI. Methods. This comprehensive narrative review synthesises mechanistic, preclinical, and clinical evidence on rifampicin across the three major osteoarticular infection types and by causative pathogen. PubMed/MEDLINE, Embase, Scopus, and the Cochrane Library were searched (January 1990–June 2026); of several hundred candidate records identified, the 101 most relevant were selected for narrative synthesis. Results. The strongest evidence supports rifampicin in staphylococcal PJI managed with debridement, antibiotics, and implant retention (DAIR), with fluoroquinolones as optimal companions; monotherapy invariably selects resistant mutants. This benefit is expected to extend beyond PJI to other osteoarticular infections managed with hardware retention—fracture-related infection and implant-associated osteomyelitis—given the shared biofilm target on retained metalwork. Benefit is attenuated in implant-exchange procedures, and evidence for *streptococci*, *Cutibacterium acnes*, and *enterococci* remains insufficient for firm recommendations. Conclusions. Rifampicin remains a valuable, difficult-to-replace agent in staphylococcal PJI treated with DAIR, provided it is introduced after surgical source control and combined with a fluoroquinolone. Observational data suggest possible benefit from introduction beyond 5 days post-debridement and courses of at least 14 days, but each threshold rests on a single study and requires confirmation. High-quality randomised trials targeting others OAI with implant retention, non-staphylococcal pathogens, optimal dosing, and treatment duration are needed.

## 1. Introduction

Osteoarticular infections represent some of the most serious complications of modern orthopaedic surgery. Whether presenting as prosthetic joint infection (PJI), fracture-related infection (FRI), or acute or chronic osteomyelitis, these conditions carry substantial morbidity, prolong hospitalisation, and generate considerable healthcare expenditure [1,2,3]. PJI occurs in 0.5–2% of arthroplasties and constitutes the leading cause of prosthetic revision in several series [1,2,3]. FRI, a complication of trauma surgery, compromises bone healing and may progress to chronic osteomyelitis with permanent functional impairment [4,5,6,7].

The unifying and defining pathogenic feature of these infections is bacterial biofilm formation on implant surfaces and bone sequestra. Biofilm confers remarkable tolerance to antibiotics and host immune defences—up to 1000-fold greater than planktonic bacteria—explaining the chronicity and high relapse rates of these infections despite adequate surgical and antibiotic treatment [8,9].

Among available antimicrobials, rifampicin occupies a singular position: it exhibits the lowest minimum biofilm eradication concentration (MBEC) against staphylococci—the predominant osteoarticular infection (OAI) pathogen [10]. Used since the 1980s in combination regimens for orthopaedic device-related infections, rifampicin was established as a cornerstone of therapy by the landmark randomised trial of Zimmerli et al. [11] and subsequently endorsed in international guidelines [1,12]. It nonetheless remains controversial owing to rapid resistance emergence in monotherapy, numerous drug–drug interactions, and an evidence base that, though growing, remains insufficiently robust for many indications.

This review aims to: (1) summarise the pharmacological properties and anti-biofilm mechanism of rifampicin; (2) review clinical data by OAI type (PJI, osteomyelitis, and FRI); (3) analyse efficacy by key pathogen; and (4) discuss optimal antibiotic combinations, alternatives, and future therapeutic directions.

## 2. Materials and Methods

### Literature Search Strategy

PubMed/MEDLINE, Embase, Scopus, and the Cochrane Library were searched from January 1990 to June 2026 using combinations of the terms “rifampicin” or “rifampin” with “osteoarticular infection”, “prosthetic joint infection”, “periprosthetic joint infection”, “osteomyelitis”, “fracture-related infection”, “biofilm”, or “debridement antibiotics and implant retention”. Searches were restricted to English- and French-language publications in humans or relevant preclinical models. Reference lists of retrieved articles, recent systematic reviews, and international guidelines were hand-searched to identify additional eligible studies. Randomised controlled trials, systematic reviews and meta-analyses, prospective and retrospective observational cohorts, and landmark preclinical (in vitro and animal) studies were prioritised; conference abstracts without a peer-reviewed full text, non-English/French reports, and studies not reporting rifampicin-specific outcomes were excluded. This strategy identified several hundred candidate records, of which the 101 judged most relevant are cited throughout this review. As with most narrative reviews, study selection was not governed by a pre-registered protocol or PRISMA flow diagram, and no formal risk-of-bias or GRADE certainty assessment was performed. Throughout this review, the level of evidence underlying each statement (randomised trial, meta-analysis, large observational cohort, small case series, or preclinical/in vitro data) is specified wherever a study is cited, so that the reader can weigh the strength of the supporting evidence; recommendations resting predominantly on observational or preclinical data are flagged as such and framed with corresponding caution rather than as firm practice statements.

## 3. Results

### 3.1. Mechanism of Action and Pharmacological Properties

#### 3.1.1. Molecular Mechanism of Action

Rifampicin belongs to the rifamycin class. It specifically inhibits bacterial DNA-dependent RNA polymerase by binding to the β-subunit encoded by rpoB, thereby blocking transcription initiation. Unlike most antibiotics that require active bacterial metabolism, rifampicin retains bactericidal activity against stationary-phase (“dormant”) bacteria—a property of critical importance in biofilms, where metabolically quiescent subpopulations coexist with actively replicating cells.

A recently elucidated mechanism [13] explains rifampicin’s superior activity against biofilms of *Staphylococcus aureus* agr mutants—those linked to the most severe biofilm-associated infections. These mutants carry an Agr-controlled rifampicin efflux system that paradoxically renders them less resistant to rifampicin than to other antibiotics, enabling enhanced biofilm penetration and killing. The clinical stakes of this resistance are considerable: in a Korean cohort of prosthetic joint infections, rpoB mutations were frequently identified among rifamycin-non-susceptible *Staphylococcus* isolates [14], and in a separate single-centre cohort of 238 patients, treatment failure was up to four times more likely in infections caused by rifampicin-resistant organisms than in those caused by rifampicin-sensitive pathogens [15].

#### 3.1.2. Pharmacokinetic Properties

Rifampicin exhibits a particularly favourable pharmacokinetic profile for OAI treatment. Oral bioavailability is 60–80%, reduced by approximately 30% when administered with food; hence, the drug should be taken during fasting. Peak plasma concentrations are reached within 2–4 h; plasma protein binding is approximately 80%. The large volume of distribution enables excellent tissue penetration including in the bone, joints, macrophages, and osteoblasts. Primary elimination is biliary via hepatic deacetylation, with a half-life of 2–5 h that shortens due to auto-induction of hepatic enzymes over the first weeks of treatment.

Bone and joint rifampicin concentrations substantially exceed the MIC values of major OAI pathogens [16]. After a 300 mg dose, peak plasma levels reach approximately 4 µg/mL, well above the MIC ranges for *S. aureus* (0.008–0.15 µg/mL) and *S. epidermidis* (0.004–0.15 µg/mL). The 300 mg twice-daily (BD), 450 mg BD, and 600 mg once-daily (OD) regimens are pharmacodynamically equivalent.

#### 3.1.3. Enzyme Induction and Drug–Drug Interactions

Rifampicin is a potent inducer of cytochrome P450 isoenzymes (CYP2B6, CYP2C8, CYP2C9, CYP2C19, CYP3A4/5/7), leading to clinically significant drug–drug interactions (DDIs). Affected drug classes include anticoagulants (warfarin, direct oral anticoagulants [DOACs]), oral contraceptives, immunosuppressants (tacrolimus, ciclosporin, sirolimus), antiretrovirals (protease inhibitors, NNRTIs, INSTI), and several co-administered antibiotics including doxycycline, linezolid, trimethoprim–sulfamethoxazole, clindamycin, and fusidic acid. These interactions do not invariably translate into clinical failure but necessitate rigorous review of co-medications before initiating rifampicin.

#### 3.1.4. Adverse Effects

The incidence of adverse effects varies across cohorts (5–30%). The most common are gastrointestinal toxicity (nausea, vomiting, diarrhoea), hepatotoxicity (particularly with pre-existing liver disease or concomitant hepatotoxic drugs, alcohol), hypersensitivity reactions (rash, fever, flu-like syndrome especially with intermittent dosing), and benign orange discolouration of urine and body fluids. Drug-related adverse events were significantly more frequent in the rifampicin group compared with controls in a large Chinese multicentre study [17] (31.65% vs. 8.78%, *p* < 0.001), leading to treatment discontinuation in 8–18% of patients across cohorts. Doses > 10 mg/kg/day are associated with poorer tolerability without improvement in success rates [18]. Beyond these general figures, rifampicin is consistently reported as the single antibiotic most often associated with adverse events in combination regimens for OAI and gastrointestinal intolerance—particularly loss of appetite—rather than nausea alone is often the dominant complaint in clinical practice; anorexia is frequently most pronounced in the first weeks of therapy, to which a subset of patients gradually accommodate, but it can lead to clinically relevant weight loss and merits proactive nutritional monitoring, especially in frail or malnourished patients. Benign orange discolouration of urine, sweat, tears, and other body fluids, while harmless, should be anticipated and explained to patients in advance to support adherence. Although daily doses ranging from 600 to 1200 mg do not appear to alter treatment success or the risk of selecting rifampicin-resistant organisms, this dose range does influence tolerability, and dose reduction or splitting (e.g., twice-daily rather than once-daily dosing) is a reasonable first step when gastrointestinal intolerance occurs, before considering discontinuation. A clinically relevant, and likely underappreciated, positive counterpart to this adverse-effect profile is that rifampicin-containing regimens are associated with a significantly reduced risk of healthcare-associated *Clostridioides difficile* infection during prolonged antibiotic therapy for OAI, presumably reflecting its distinct spectrum and biliary elimination [19]. In practice, before initiating rifampicin, we recommend a systematic review of co-medications for interacting drug classes, baseline and periodic monitoring of liver function tests (e.g., at baseline, 2–4 weeks, and thereafter if the patient is symptomatic or has pre-existing liver disease), and explicit counselling on expected gastrointestinal effects and urine discolouration to support early recognition and adherence rather than unplanned discontinuation.

### 3.2. Bacterial Biofilm and Anti-Biofilm Activity of Rifampicin

#### 3.2.1. Biofilm Formation and Mechanisms of Antibiotic Tolerance

**Figure 1:**
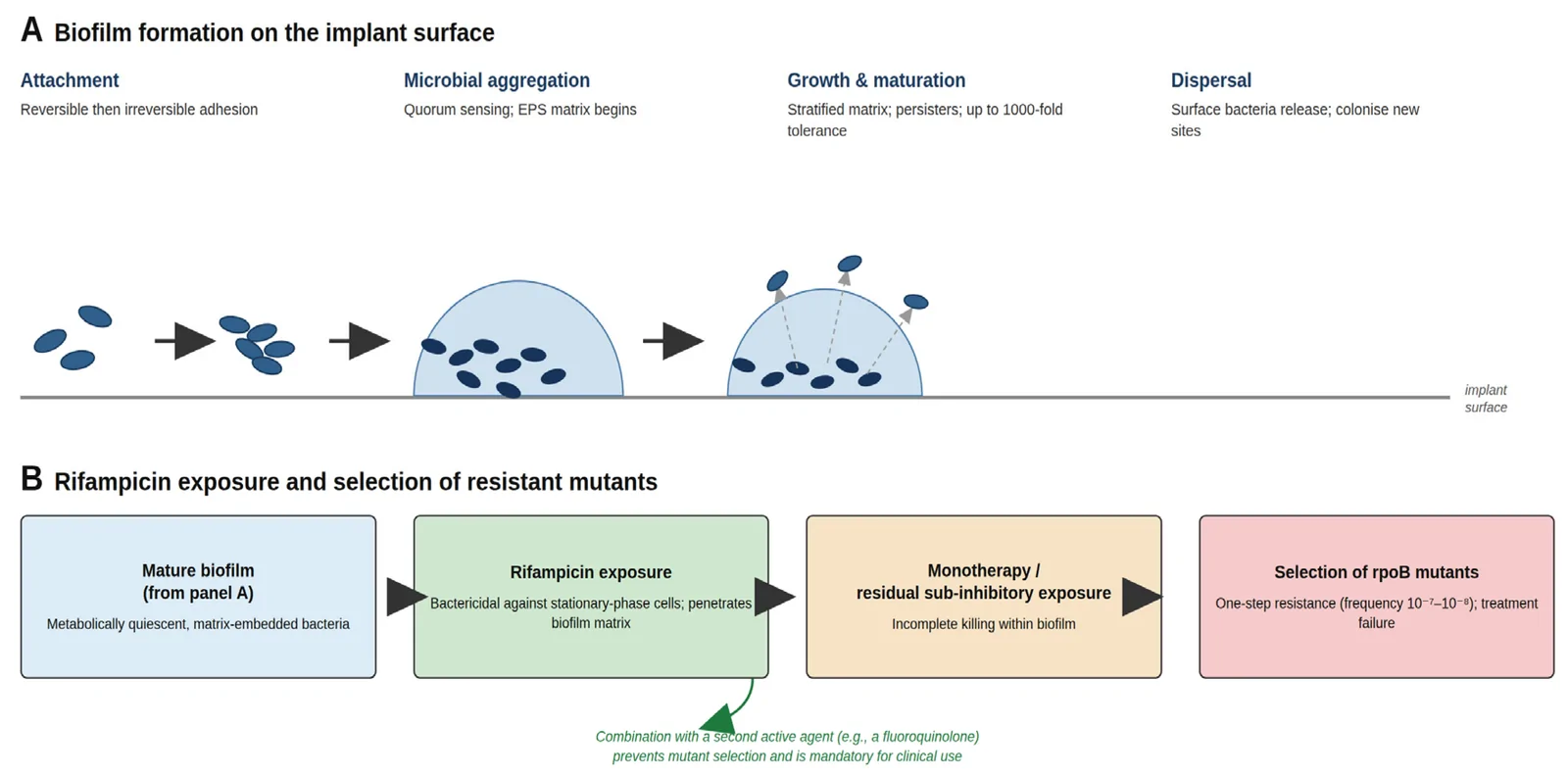
Biofilm formation and selection of rifampicin-resistant mutants. (**A**) Stages of biofilm development on an implant surface: reversible then irreversible attachment of planktonic bacteria, microbial aggregation with quorum-sensing-driven extracellular polymeric substance (EPS) production, growth and maturation into a stratified, matrix-embedded community containing metabolically quiescent persister subpopulations (conferring tolerance up to 1000-fold that of planktonic bacteria), and dispersal of surface bacteria to colonise new sites. (**B**) Within a mature biofilm, rifampicin is bactericidal against stationary-phase bacteria and penetrates the biofilm matrix; however, if used as monotherapy or if exposure is sub-inhibitory, incomplete killing allows selection of rpoB mutants (one-step resistance frequency 10^−7^–10^−8^), leading to treatment failure. Combination with a second active agent (e.g., a fluoroquinolone) prevents mutant selection and is mandatory for clinical use (Section 3.5).

Biofilm is defined as a structured microbial community embedded in a self-produced extracellular polymeric matrix and attached to a surface. Formation proceeds in three stages: initial adhesion to the implant surface, accumulation and maturation with matrix production, and dispersal (Figure 1). Once established, biofilm confers antibiotic tolerance up to 1000-fold greater than planktonic bacteria [8]. Multiple mechanisms underlie this tolerance: impaired penetration of certain antibiotics through the stratified biofilm matrix; metabolically quiescent “persister” subpopulations surviving otherwise bactericidal concentrations; and altered microenvironment featuring anaerobic niches, pH gradients, and osmotic shifts. The minimum biofilm eradication concentration (MBEC) is therefore a more clinically relevant metric than the standard planktonic MIC.

#### 3.2.2. In Vitro Anti-Biofilm Activity

Multiple in vitro studies consistently demonstrate that rifampicin achieves the lowest MBEC values against staphylococcal biofilms compared with other available antibiotics (Supplementary Table S1). Molina-Manso et al. compared nine antibiotics against 32 *S. aureus* and *S. epidermidis* strains from PJI patients: while no antibiotic achieved complete eradication alone, rifampicin and tigecycline exhibited the lowest MBECs [10].

Tang et al. demonstrated significant synergy (≥100-fold reduction in bacterial burden) between rifampicin and linezolid, fosfomycin, minocycline, fusidic acid, and tigecycline against MRSA biofilms, with the combinations also limiting emergence of resistant mutants—in stark contrast to rifampicin monotherapy [20].

Alagboso et al. showed that at 8 µg/mL, rifampicin eradicated intracellular *S. aureus* from osteoblasts and restored extracellular matrix mineralisation at 21 days, suggesting dual antibacterial and osteoprotective benefits [21]. These favourable in vitro results should not, however, be over-generalised to every companion agent: using a 5-day mature *S. aureus* biofilm model, Boot et al. found that adding rifampin at a peri-implant-relevant concentration did not improve, and at the highest gentamicin concentration tested even slightly worsened, biofilm eradication compared with gentamicin alone, across continuous, intermittent, and burst-release exposure profiles [22]. This finding is a useful reminder that rifampicin’s anti-biofilm benefit is not universal across every partner drug or exposure scenario and is best documented for the fluoroquinolone- and cell-wall-active partner combinations discussed in Section 3.5, rather than for aminoglycosides delivered locally.

#### 3.2.3. Animal Models

Preclinical evidence underpinning rifampicin use in OAI spans five decades. Collectively, in vivo studies have established: the superiority of rifampicin-containing combinations over monotherapy; the importance of companion drug selection; the risk of resistance emergence with monotherapy; and dose-dependent pharmacodynamic relationships.

##### Guinea Pig Tissue-Cage Models (Foundational Evidence)

Zimmerli et al. established the subcutaneous tissue-cage model in guinea pigs as the canonical system for foreign-body infection research [12]. Rifampicin-containing combinations consistently achieved bacterial eradication whereas monotherapy and non-rifampicin combinations failed. With four doses of rifampicin, implant-associated *S. aureus* infection was cured in 100% of tissue cages when therapy was initiated within 12 h of inoculation. The model confirmed that rifampicin monotherapy invariably selects for resistant mutants (frequency 10^−7^ to 10^−8^), establishing the absolute requirement for combination therapy. These guinea pig data formed the experimental basis for the landmark RCT published in JAMA in 1998. It is worth noting, however, that the clinical foundation for the current rifampicin combination dogma rests disproportionately on the single, comparatively small randomised trial that followed (Section 3.3.1), whose control arm (ciprofloxacin monotherapy) is no longer an agent any group would use as sole antistaphylococcal therapy today; no subsequent randomised trial has reproduced comparable success for a rifampicin combination against a genuinely anti-staphylococcal comparator such as an active fluoroquinolone or beta-lactam alone, and this near-universal recommendation may reasonably be challenged as ongoing non-inferiority trials such as RiCOTTA [23] report their results (Section 3.8.2); this tension between longstanding clinical practice and a comparatively narrow trial base has been explicitly debated in the literature [24].

##### Murine Models

Several murine implant-associated osteomyelitis models confirmed and extended the guinea pig findings. In a well-designed murine K-wire osteomyelitis model, rifampicin-containing combinations (rifampicin + vancomycin; rifampicin + linezolid) achieved eradication whereas combinations of two non-rifampicin antibiotics (vancomycin + linezolid; vancomycin + daptomycin) were not more active than single agents—confirming that the anti-biofilm effect is specific to rifampicin and cannot be recapitulated by combining other agents [25]. Biofilm formation reduced susceptibility 500–2000-fold relative to planktonic bacteria. Bioluminescent imaging studies by Niska et al. further demonstrated that six weeks of vancomycin + rifampicin substantially outperformed vancomycin alone in a mouse knee PJI model, with resistance emerging in the monotherapy but not the combination arm [26,27].

##### Rat and Rabbit Osteomyelitis Models

In a rat model of MRSA osteomyelitis, rifampicin monotherapy achieved a median tibial bacterial load of 0.1 log_10_ CFU/g (vs. 6.04 log_10_ CFU/g in controls and 4.81 log_10_ CFU/g with vancomycin alone); rifampicin combined with vancomycin or omadacycline yielded no detectable MRSA. Resistance emerged in three of 16 monotherapy animals and in none receiving combination therapy. Cercenado et al. demonstrated that ceftaroline combined with rifampicin sterilised bone in significantly more animals than either drug alone in a rat MRSE osteomyelitis model without implant [28].

##### PET-Guided Pharmacodynamic Modelling

A landmark translational study by Gordon et al. used dynamic ^11^C-rifampicin PET imaging in patients and a murine *S. aureus* implant-infection model to characterise rifampicin pharmacokinetics at the infection site [29]. Bone tissue concentrations were substantially lower than plasma concentrations at standard doses. High-dose rifampicin (35 mg/kg once daily in mice) increased bone concentrations approximately 10-fold and achieved significantly greater bacterial killing. Four weeks of high-dose combination therapy was equivalent to a standard six-week regimen—suggesting that pharmacodynamically optimised dosing could shorten treatment duration.

##### Recalcitrant PJI and Novel Delivery Systems

Irwin et al. developed a rat model of recalcitrant PJI using biofilm inocula to replicate established implant biofilm: rifampicin addition to gentamicin reduced the MBEC_60_ 8-fold (1024 → 128 µg/mL), confirming synergy at the biofilm level in vivo [30]. In a porcine one-stage exchange model, Vittrup et al. found no significant microbiological or histopathological improvement with rifampicin addition to moxifloxacin [31], mirroring the clinical meta-analytic finding of attenuated rifampicin benefit in implant-exchange procedures. Sebastian et al. demonstrated that systemically administered rifampicin binds to and accumulates within locally implanted hydroxyapatite particles (“accretion”), providing proof of concept for hydroxyapatite-based rifampicin depots [32]. Gidari et al. found that in a rat MRSA wire osteomyelitis model, tedizolid + rifampicin achieved sterility in only 45% of animals versus 88% with vancomycin + rifampicin, cautioning against direct extrapolation from in vitro synergy to in vivo efficacy [33]. A recent study by Buzisa Mbuku et al. demonstrated that enzyme-enhanced antibiotic therapy reduced biofilm burden to undetectable levels in an implant-associated infection model, illustrating a promising adjunctive strategy to help overcome residual biofilm-related antibiotic tolerance [34].

### 3.3. Rifampicin Efficacy by Infection Type

#### 3.3.1. Prosthetic Joint Infections

##### Background and Microbiology

PJI is the most severe complication of joint arthroplasty. *S. aureus* and coagulase-negative staphylococci (CoNS) account for >50% of cases [3]. In a large international cohort [35], *S. aureus* represented 33.6% of PJI, CoNS 24.9%, with *Pseudomonas aeruginosa* (8.6%) and *enterococci* completing the spectrum. Surgical strategy—DAIR, one-stage, or two-stage revision—is the primary determinant of rifampicin indication.

##### Debridement, Antibiotics, and Implant Retention (DAIR)

DAIR is the clinical context with the strongest evidence for rifampicin and is the recommended first-line surgical strategy for acute PJI (<3–4 weeks of symptoms, stable implant) in current guidelines [1,3]. Four randomised controlled trials (RCTs) have been conducted, complemented by large cohort studies (Supplementary Table S2).

Zimmerli et al. [11] reported a 100% cure rate with ciprofloxacin + rifampicin versus 58% with ciprofloxacin + placebo in 69 patients with staphylococcal orthopaedic implant infections. This landmark trial is limited by a small sample size and a fluoroquinolone monotherapy comparator arm prone to resistance selection.

Karlsen et al. [36] found no significant difference in the cure rate between rifampicin combination (74%) and monotherapy without rifampicin (72%) in 99 patients (RR 1.03; 95% CI 0.73–1.45). The principal limitation is the use of oral cloxacillin as the control agent, which has poor bioavailability and limited bone penetration.

Lora-Tamayo et al. [37] conducted an open-label multicentre RCT comparing short (8 weeks) versus standard long-duration (3–6 months) levofloxacin plus rifampicin in 63 patients with acute staphylococcal PJI managed by DAIR. Per-protocol cure rates were 91.7% (short) versus 95.0% (long) (difference +3.3%; 95% CI −11.7% to +18.3%), establishing statistical non-inferiority for hip prostheses and supporting a minimum 8-week regimen when clinical response is favourable. Although the primary objective of this trial was not to assess the efficacy of rifampicin, overall treatment success rates were remarkably high, further supporting the effectiveness of rifampicin-based combination therapy in DAIR-managed prosthetic joint infections.

The multicentre observational study by Beldman et al. [38] (669 patients, six centres, four countries, 1997–2017) provides the most informative real-world data. One-year failure was 32.2% with rifampicin versus 54.2% without (*p* < 0.001). The effect was most pronounced for prosthetic knee infections. Key findings: (1) introducing rifampicin within 5 days of debridement was associated with higher failure (40.8% vs. 20.9% if >5 days post-debridement); and (2) cotrimoxazole–rifampicin combinations had the highest failure rate (38%) while clindamycin/fluoroquinolone + rifampicin had the lowest (14%).

The meta-analysis by Scheper et al. [39] (62 predominantly observational studies) calculated a pooled relative risk (RR) for rifampicin success of 1.10 (95% CI 1.00–1.22). Minimum treatment duration appears critical: Becker et al. [40] demonstrated that rifampicin for <14 days was associated with significantly higher failure risk than >14 days (*p* = 0.0017).

Among prospective cohort studies, Aboltins et al. [41] reported 90% infection-free survival at median 32-month follow-up in 20 patients with staphylococcal PJI treated with DAIR and oral rifampicin plus fusidic acid, providing early prospective validation of the rifampicin-based oral strategy. Byren et al. [42] described outcomes in 112 infected arthroplasties (18% recurrence at mean 2.3 years), with shorter courses (<3 months) associated with higher recurrence. Lora-Tamayo et al. for the REIPI group [43] reported the largest single-pathogen DAIR series at that time (345 *S. aureus* episodes), achieving 55% overall success; rifampicin-containing combinations appeared to partially homogenise outcomes between MSSA and MRSA. Tornero et al. [44] demonstrated that failure rates were significantly lower with rifampicin + fluoroquinolone (8.3%) compared with rifampicin + linezolid, cotrimoxazole, or clindamycin (27.8%), reinforcing the primacy of the rifampicin–fluoroquinolone pairing. Taken together, despite differences in design, population, and effect measures across these studies, they converge on a consistent signal favouring rifampicin combination therapy—particularly with a fluoroquinolone partner—in DAIR-managed staphylococcal PJI, although the specific timing and duration thresholds identified each rest on a single study and await confirmation in prospective trials.

The RiCOTTA trial [23] is an ongoing multicentre non-inferiority RCT evaluating antibiotic monotherapy (without rifampicin) versus rifampicin combination therapy during the oral phase of staphylococcal PJI managed by DAIR (target *n* = 316, 80% power, 10% non-inferiority margin). Its results will provide decisive evidence on the necessity of rifampicin during oral maintenance therapy.

##### One-Stage-Revision

Kobayashi et al. [45] (27 studies, 2 RCTs) demonstrated the benefit of rifampicin-containing regimens in one-stage revision (RR 1.11; 95% CI 1.03–1.20; *p* = 0.009). Kruse et al. [46] found a protective odds ratio (OR) of 0.64 (95% CI 0.43–0.95) for *S. aureus* and 0.44 (0.22–0.89) for *Cutibacterium* spp. in revision surgery. However, Kramer et al. [47] in a multicentre ESGIAI cohort (375 staphylococcal PJI, one-stage and two-stage revision) found no statistically significant benefit of rifampicin in the one-stage revision subgroup, with *S. aureus* itself identified as an independent failure predictor—a confounding factor that may partially explain the divergence with meta-analytic data. Taken together, the meta-analytic pooled estimates and the largest dedicated one-stage-revision cohort point in different directions, illustrating how heterogeneity in pathogen mix, case definitions, and unmeasured confounders such as pathogen virulence across studies can obscure a true, moderate rifampicin benefit in this surgical setting.

##### Two-Stage Revision

The meta-analysis by Yusuf et al. [48] found no significant benefit of rifampicin in two-stage procedures (success approximately 90% in both arms; limited evidence). Prats-Peinado et al. [49] showed that two-stage revision using high-dose local antibiotics in bone cement spacers achieved equivalent results without systemic rifampicin. A recent Chinese multicentre study [17] found no significant difference in remission between rifampicin and non-rifampicin groups across all surgical strategies (79.75% vs. 73.65%, *p* = 0.083). Gómez-Junyent et al. [50] (215 *S. aureus* PJI managed with implant removal) found rifampicin use did not independently predict the outcome after multivariate adjustment, suggesting surgical strategy and pathogen virulence dominate when the implant—and thus the principal biofilm target—is physically eliminated.

A nuanced picture emerges from the Kramer et al. ESGIAI cohort [47]: while no overall rifampicin benefit was identified, subgroup analysis of chronic PJIs treated by two-stage exchange demonstrated significantly lower failure in rifampicin recipients—biologically plausible given that residual sessile organisms on bone surfaces may persist during the spacer interval. The comparative meta-analysis by Kruse et al. [46] also found that rifampicin was associated with improved outcomes in patients managed with exchange arthroplasty (one- or two-stage revision), whereas no significant protective effect was observed in studies limited to implant retention (DAIR). Thus, data remain controversial; however, converging findings suggest rifampicin’s benefit is maximal when the implant is retained (DAIR)—where residual biofilm on the implant surface is the primary target. When the implant is removed in a two-stage procedure, biofilm is physically eliminated, rendering rifampicin less essential.

#### 3.3.2. Osteomyelitis

Osteomyelitis—acute or chronic, haematogenous or post-traumatic—shares with PJI the central roles of biofilm and staphylococcal predominance. The standard recommendation is 6 weeks of antibiotic therapy, initiated parenterally then transitioned to oral. Spellberg and Lipsky [51] demonstrated that oral antibiotics achieving adequate bone concentrations yield cure rates comparable to parenteral therapy, and that adjunctive rifampicin may improve outcomes, particularly for device-associated infections.

For diabetic foot osteomyelitis, Senneville et al. [52] reported an 88.2% cure rate at the end of treatment and 76.5% at the 22-month follow-up with rifampicin + ofloxacin for 6 months in 17 diabetic patients—one of the few prospective datasets in this challenging indication.

Rifampicin is incompatible with standard polymethylmethacrylate (PMMA) bone cement as it interferes with polymerisation. Alternative local delivery platforms are in development: porous calcium phosphate scaffolds loaded with vancomycin and rifampicin significantly reduced bacterial burden relative to vancomycin-PMMA [53]. An antibiotic bone void filler putty releasing vancomycin and rifampicin over 6 weeks demonstrated complete bacterial elimination and bone regeneration in a rat osteomyelitis model [54].

#### 3.3.3. Fracture-Related Infections

FRI are feared complications of trauma surgery, characterised by infection at fracture or surgical fixation sites, frequently involving biofilm-forming pathogens [4,5,6,7]. Management requires a structured multidisciplinary approach combining orthopaedic surgery, infectious diseases, and microbiology expertise. Key surgical elements include thorough debridement with normal saline irrigation, fracture stability (absolute priority for bone healing), dead space management, and adequate soft-tissue coverage. With infection duration <3–4 weeks, implant retention achieves success rates of 86–100%. Rifampicin’s role in FRI is largely extrapolated from the PJI literature rather than independently established (Supplementary Table S3). The meta-analysis by Kobayashi et al. [45] included spinal instrumentation infections and showed the benefit of rifampicin-based regimens in DAIR-equivalent procedures on osteosynthesis hardware, and Kruse et al. [46] found a protective effect of rifampicin-containing regimens in non-DAIR-exclusive surgical strategies (OR 0.47; 95% CI 0.27–0.80). McNally et al. [55] showed that local antibiotic implantation—not rifampicin specifically—reduced FRI recurrence from 18.7% to 10.0% (HR 0.48; 95% CI 0.29–0.81).

However, dedicated FRI cohorts have not confirmed an independent benefit of rifampicin. In the largest reported FRI-DAIR cohort, Buijs et al. [56] administered rifampicin to 65% of patients but did not find rifampicin use to be an independent predictor of treatment success on multivariable analysis, suggesting that the benefit observed in PJI does not translate directly to the fracture-fixation setting, where hardware geometry, soft-tissue compromise, and fracture-healing requirements differ substantially. Consistent with this, the multicentre EAT-FRI study (Corrigan et al. [57]) found that non-compliance with guideline-based antimicrobial therapy—including incorrect use or non-use of rifampicin—was not associated with worse clinical outcomes (treatment failure 12.1% compliant vs. 13.2% non-compliant, *p*  =  0.87), further questioning whether rifampicin exerts the same magnitude of effect in FRI as in PJI.

Taken together, the current evidence supports a pragmatic, extrapolated role for rifampicin in FRI rather than an independently proven one. It may be considered as an adjunct to surgical debridement and hardware stabilisation—not a substitute for source control—in patients with retained fixation devices, a confirmed rifampicin-susceptible staphylococcal infection, and adequate soft-tissue coverage, following the same principles established for PJI: introduction only after adequate surgical source control, combination with a companion agent (never monotherapy), and a sufficient treatment duration. Rifampicin use should never delay or substitute for adequate surgical source control, which remains the primary determinant of outcome in FRI.

### 3.4. Pathogen-Specific Analysis

#### 3.4.1. *Staphylococcus aureus* (MSSA and MRSA)

*S. aureus* is the primary OAI pathogen, responsible for 33–49% of PJI and >50% of osteomyelitis cases. Its virulence, biofilm capacity, and metastatic potential make it the principal target organism for rifampicin-based therapy [3]. The strongest data concern staphylococcal PJI treated with DAIR (Supplementary Table S4). Kobayashi et al. [45] demonstrate significant benefit for *S. aureus* infections (RR 1.27; 95% CI 1.11–1.45; *p* < 0.001). Kruse et al. [46] find an OR for failure of 0.64 (95% CI 0.43–0.95; *p* = 0.03). Scheper et al. [39] report pooled success rates of 69% for the hip versus 54% for the knee *S. aureus* PJI.

A large claims database study [58] (52,588 hip arthroplasty patients) confirmed that rifampicin combination therapy was associated with a significantly reduced risk of revision surgery in staphylococcal PJI managed with implant retention (OR 0.61; 95% CI 0.47–0.80; *p* < 0.001). Methicillin susceptibility does not significantly influence prognosis [39]: MSSA and MRSA achieve comparable success rates under rifampicin provided the companion antibiotic is appropriately matched (fluoroquinolone for MSSA; glycopeptide or linezolid for MRSA with quinolone resistance).

Rifampicin resistance drastically worsens prognosis. Krizsan et al. [59] report cure rates of 92.5% for susceptible versus 60.0% for resistant strains after two-stage revision. Auñón et al. [60] show that rifampicin resistance and MDR/XDR status are independent failure predictors; double resistance to levofloxacin and rifampicin carries the strongest failure association (OR 8.01; 95% CI 2.82–22.77; *p* < 0.001).

These findings further illustrate the poorer outcomes associated with infections caused by multidrug-resistant pathogens or following previous antimicrobial exposure, which may have selected for resistant organisms.

Leal et al. [61] evaluated 87 patients meeting IDSA criteria for rifampicin therapy: those completing a full rifampicin course achieved 71.4% treatment success; patients unable to receive rifampicin due to DDIs were significantly less likely to achieve cures.

#### 3.4.2. Coagulase-Negative Staphylococci (CoNS)

CoNS—principally *S. epidermidis*, *S. capitis*, *S. lugdunensis*, *S. warneri*, *S. hominis*, and *S. haemolyticus*—account for approximately 25% of PJI. Over 50% are methicillin-resistant; primary rifampicin resistance reaches 20% in some cohorts [62]. CoNS form particularly resistant biofilms, explaining their predilection for implant-associated infections. The Scheper meta-analysis [39] demonstrates statistically superior pooled success rates for CoNS PJI (hip 83%, knee 73%) versus *S. aureus*, with a positive association with rifampicin use. Rifampicin resistance in CoNS involves rpoB mutations: Edslev et al. [63] identified 64 (6.8%) resistant isolates among 940 *S. epidermidis* PJI strains, with 11 non-synonymous mutations, several previously undescribed.

#### 3.4.3. *Streptococci*

*Streptococci* account for 9–10% of PJI, predominantly *Streptococcus agalactiae* (group B), *S. dysgalactiae* (group C/G), and viridans *streptococci* [3]. In vitro, most *streptococcal* species exhibit low MIC values for rifampicin and levofloxacin. However, in a comparative in vitro study of rifampin, rifabutin, and rifapentine against staphylococcal, *streptococcal*, and *enterococcal* isolates from PJI, anti-biofilm activity was significantly lower against *streptococcal* and *enterococcal* biofilms than against staphylococcal biofilms, with the exception of some *Streptococcus mitis* group isolates, underscoring that rifampicin’s biofilm-active reputation is largely a *staphylococcus*-specific phenomenon [64]. Penicillins remain first-line agents but have poor oral bioavailability and no activity against stationary-phase bacteria.

Clinical evidence is limited and contradictory (Supplementary Table S5). Fiaux et al. [65], in a retrospective multicentre cohort study (95 patients, four French centres), reported 70.5% overall remission. Univariate analysis suggested a trend favouring rifampicin + levofloxacin, but multivariate analysis identified only prosthesis location (hip vs. knee) as an independent prognostic factor—not rifampicin. In a multicentre cohort of 462 *streptococcal* PJI [66], early rifampicin use was a protective factor per treatment day (HR 0.98/treatment-day in the first 30 days), but was not independently significant in multivariate analysis, suggesting surgical factors dominate. Borens et al. [67] (single-centre, retrospective) found *streptococcal* PJI failure rates similar to that of other aetiologies (23% vs. 21%) with no independent rifampicin effect.

The most comprehensive meta-analysis of *streptococcal* DAIR (Gerritsen et al. [68], 1367 patients, 25 studies) found pooled DAIR success of 71% with no clear rifampicin benefit after adjustment, while confirming superior outcomes for knee over hip PJI. Meta-analyses conflict: Kobayashi et al. [45] show significant benefit (RR 1.18; 95% CI 1.02–1.38; *p* = 0.03); Yusuf et al. [48] and Gerritsen et al. [68] find no significant benefit. Current evidence is insufficient for a firm recommendation.

#### 3.4.4. *Cutibacterium acnes*

*Cutibacterium acnes* (formerly *Propionibacterium acnes*) is responsible for approximately 10% of PJI, predominantly shoulder and hip arthroplasties. Its indolent clinical presentation frequently delays diagnosis. Furustrand Tafin et al. [69] demonstrated in vitro superiority of rifampicin-containing combinations over monotherapy against *C. acnes* biofilms. Clinical results are, however, inconsistent. Jacobs et al. [70] (60 patients, monocentre) reported 93% success at 1 year and 86% at 2 years with no significant difference between rifampicin and non-rifampicin groups. Kusejko et al. [71] (187 patients, nine countries, median 36-month follow-up) found no impact of rifampicin on outcome after confounding adjustment; only DAIR strategy and treatment duration <6 weeks predicted failure.

Meta-analyses conflict: Kobayashi et al. [45] show significant benefit (RR 1.11; 95% CI 1.01–1.22; *p* = 0.03); Kruse et al. [46] find OR for failure of 0.44 (0.22–0.89); and Saltiel et al. [72] show no benefit. Inherently high cure rates in *C. acnes* infections may mask any marginal rifampicin benefit. The international RIFACute trial [73] (NCT05902221, initiated 2023, estimated completion 2027) will specifically evaluate rifampicin impact in *C. acnes* PJI.

#### 3.4.5. *Enterococci*

*Enterococci*—principally *E. faecalis* and *E. faecium*—are encountered in PJI, particularly at the hip. Treatment is complicated by intrinsic resistance patterns and biofilm persistence. In vitro, Holmberg et al. [74] evaluated biofilm susceptibility in *E. faecalis* from prosthetic joint infections: ciprofloxacin + rifampicin followed by linezolid + rifampicin was the most effective regimen, with less resistance emergence than monotherapy or ampicillin combinations. In a retrospective multinational study, Tornero et al. [75] demonstrated that rifampicin-based combination therapy was associated with a significantly lower treatment failure rate than alternative antimicrobial regimens in early prosthetic joint infections diagnosed within 30 days after arthroplasty. These findings warrant confirmation in larger prospective studies. Clinical data are extremely limited and no randomised trial is available for *enterococcal* OAI.

### 3.5. Antibiotic Combinations with Rifampicin

Rifampicin must never be used as monotherapy in OAI owing to rapid one-step resistance emergence via rpoB mutation (Figure 1). Combination with a second antibiotic is mandatory. The choice of companion drug is a critical determinant of clinical outcome (Supplementary Table S6). Because rifampicin’s enzyme-inducing effect (Section 3.1.3) lowers plasma concentrations of most companion agents rather than raising them, the practical implication is generally the opposite of dose reduction: where feasible, the companion drug should be dosed at the higher end of its usual range and its dosing schedule stabilised before rifampicin is introduced, rather than assuming that rifampicin’s excellent oral bioavailability and tissue penetration permit a lower companion dose; specific dose-adjustment thresholds have not, however, been formally validated and are discussed per combination below.

#### 3.5.1. Rifampicin + Fluoroquinolones—Reference Combination

The rifampicin–fluoroquinolone combination is the most extensively studied regimen. Zimmerli et al. [11] established it for staphylococcal implant infections. Kobayashi et al. [45] confirm that the positive impact of rifampicin is statistically significant only when combined with a fluoroquinolone. Beldman et al. [38] report 14% failure with fluoroquinolone + rifampicin versus 38% with cotrimoxazole + rifampicin.

Levofloxacin is preferred over ciprofloxacin for staphylococci owing to its lower MIC; moxifloxacin has the lowest MIC against MSSA and intracellular activity but co-administration with rifampicin reduces moxifloxacin AUC_0–24_ by 67–85% in target tissue compartments [76,77]. Rifampicin significantly reduces fluoroquinolone tissue concentrations through CYP450 and P-glycoprotein induction; residual concentrations likely remain above pathogen MIC breakpoints. It is recommended to establish stable fluoroquinolone dosing before introducing rifampicin. In non-staphylococcal Gram-positive OAI, a moxifloxacin–rifampicin regimen has also been reported with favourable outcomes in a small series [78].

#### 3.5.2. Rifampicin + Clindamycin

Clindamycin offers excellent oral bioavailability, anti-biofilm and anti-adhesion activity, and good bone and joint penetration. Rifampicin reduces clindamycin serum concentrations [79] through CYP450 induction. In a pharmacokinetic study including 34 patients, Bernard et al. [79] demonstrated that co-administration of rifampicin resulted in a marked reduction in clindamycin plasma concentrations, frequently falling below the therapeutic range, although no significant impact on clinical outcomes was observed. Leijtens et al. [80] report 94% cure with clindamycin + rifampicin. Czekaj et al. [81] confirm good results in small series. Beldman et al. [38] show 14% failure with clindamycin + rifampicin—comparable to the fluoroquinolone + rifampicin combination. In contrast, in a retrospective cohort of 143 patients, Tornero et al. [44] reported significantly higher treatment failure rates with the rifampicin–clindamycin combination than with rifampicin–levofloxacin (27.8% vs. 8.3%, respectively). Overall, despite the marked pharmacokinetic interaction resulting in reduced clindamycin exposure, the available clinical evidence generally supports the efficacy of the rifampicin–clindamycin combination. Therefore, this regimen appears to represent a reasonable alternative when fluoroquinolones cannot be combined with rifampicin because of resistance, intolerance, contraindications, or safety concerns.

#### 3.5.3. Rifampicin + Beta-Lactams

The absence of significant pharmacokinetic interaction between rifampicin and beta-lactams is an advantage. Beta-lactams active against staphylococci (flucloxacillin, oxacillin for MSSA; ertapenem for MRSA) can be combined with rifampicin. In a retrospective multinational study, Tornero et al. [44] likewise observed comparable efficacy between rifampicin–amoxicillin and rifampicin–fluoroquinolone combination therapies. Corts-Penfield et al. [82] suggested in a retrospective study that beta-lactam monotherapy was not inferior to beta-lactam + rifampicin combination in DAIR-treated PJI. However, their limitations—poor oral bioavailability, limited bone penetration, no activity against stationary-phase bacteria—make them less ideal as long-term oral maintenance partners.

#### 3.5.4. Rifampicin + Cotrimoxazole

Trimethoprim–sulfamethoxazole is frequently used in some MRSA or *streptococcal* OAI. Two small randomised controlled trials reported encouraging results for the cotrimoxazole–rifampicin combination. Nguyen et al. [83] demonstrated comparable cure rates between rifampicin–linezolid and rifampicin–trimethoprim/sulfamethoxazole combinations (89.3% vs. 78.6%, *p* = 0.47), while Euba et al. [84] reported similar cure rates with an oral rifampicin–trimethoprim/sulfamethoxazole regimen and a standard flucloxacillin-based regimen in patients with chronic osteomyelitis. However, larger observational studies have yielded less favourable results. In a retrospective multinational cohort, Tornero et al. [44] reported higher treatment failure rates with the rifampicin–trimethoprim/sulfamethoxazole combination than with rifampicin–fluoroquinolone or rifampicin–amoxicillin regimens. Similarly, a large cohort by Beldman et al. [38] observed a 38% treatment failure rate with rifampicin–trimethoprim/sulfamethoxazole, significantly higher than with other rifampicin-based combinations. Taken together, although small randomised studies suggest that rifampicin–trimethoprim/sulfamethoxazole may be effective, the less favourable outcomes reported in larger cohorts indicate that this combination should generally be reserved as an alternative companion regimen when other options are not feasible.

#### 3.5.5. Rifampicin + Linezolid

Because of its excellent oral bioavailability and broad spectrum of activity against Gram-positive cocci, linezolid represents an attractive companion agent for rifampicin in the treatment of orthopaedic implant-associated infections caused by MRSA or multidrug-resistant Gram-positive pathogens, particularly *Enterococcus faecium*. Kobayashi et al. [45] show a trend towards more failures with rifampicin + linezolid; Tornero et al. [44] confirm it is the combination most independently associated with higher failure rates. Rifampicin-induced reduction in linezolid concentrations likely underlies these results. Morata et al. [85] reported 79.9% overall cure with linezolid ± rifampicin and also demonstrated selective utility in patients with fluoroquinolone- and/or rifampicin-resistant staphylococci when no viable alternatives exist. However, combining rifampicin with linezolid is not generally advisable given this drug–drug interaction.

#### 3.5.6. Rifampicin + Fusidic Acid

Fusidic acid (available in Europe) achieves good bone penetration. Drancourt et al. [86] compared rifampicin + fusidic acid versus rifampicin + ofloxacin for staphylococcal implant infections: success rates of 55% versus 50%, respectively, with no significant difference. A phase 2 RCT was terminated prematurely owing to significant pharmacokinetic interactions between the two agents.

#### 3.5.7. Rifampicin + Doxycycline

Doxycycline offers broad Gram-positive spectrum, excellent bioavailability, and good tissue penetration, but its concentration is reduced by rifampicin. Clinical data in OAI are extremely limited. Doxycycline is used primarily as long-term suppressive therapy when complete eradication is not achievable (inoperable patients, persistent biofilm).

### 3.6. Dosing and Timing of Rifampicin

#### 3.6.1. Dosing Regimens

Rifampicin dosing varies across studies and guidelines, ranging from 300 mg BID or TID, 450 mg BD to 600 mg OD (rarely 600 mg BD). Pharmacodynamic data and clinical studies converge to suggest that 300 mg BD and 600 mg OD are pharmacologically equivalent for *S. aureus* and *S. epidermidis*. Beldman et al. [38] found no efficacy difference between 600 mg/day as a single dose and 450 mg BD. Data indicate that doses > 10 mg/kg/day are associated with poorer tolerability without improvement in success rates [18]. Rifampicin should be taken during fasting, as food reduces absorption by approximately 30%. Rifampicin’s oral bioavailability also facilitates ambulatory management, including outpatient parenteral antimicrobial therapy (OPAT) pathways for the companion agent, with favourable efficacy, tolerability, and cost outcomes reported in chronic osteomyelitis [87].

These dosing conventions may not be pharmacodynamically optimal at the infection site. Gordon et al. [29] demonstrated using ^11^C-rifampicin PET imaging that bone tissue rifampicin concentrations at standard oral doses were substantially lower than plasma concentrations, and that high-dose rifampicin achieved 10-fold higher bone concentrations with significantly enhanced bacterial killing. Four weeks of high-dose combination therapy achieved outcomes equivalent to a standard six-week regimen—arguing for individualised dosing guided by tissue-level pharmacodynamic targets rather than plasma concentrations alone.

#### 3.6.2. Timing of Introduction

Timing of rifampicin introduction is critically important. Evidence consistently supports delaying rifampicin until surgical source control has been achieved and bacterial burden reduced by debridement. Beldman et al. [38] report failure rates of 40.8% when rifampicin was introduced within 5 days of debridement versus 20.9% when introduced thereafter (*p* < 0.001). Dessert et al. [88], in a broader cohort of staphylococcal implant infections (not limited to PJI), similarly found on multivariate analysis that neither the delay, dose, nor duration of rifampicin was independently associated with clinical failure or resistance emergence, again highlighting that dedicated RCTs are needed to resolve this question definitively. In practice, a pragmatic approach is to introduce rifampicin only once surgical drains have been removed and there is no further observable wound drainage, typically 3–5 days after debridement, since starting rifampicin too early against a still-high bacterial burden increases the risk of selecting resistant mutants [89].

#### 3.6.3. Optimal Duration

The optimal duration of rifampicin therapy remains uncertain. Scheper et al. [90] showed in a large observational cohort that a short-course rifampicin strategy (5 days, combined with clindamycin or flucloxacillin) was as effective as traditional long-term combination therapy (adjusted HR for failure = 1.21; 95% CI 0.34–4.40). Conversely, in a large multicentre retrospective study of acute staphylococcal PJIs treated with DAIR, Becker et al. [40] identified rifampicin treatment lasting <14 days as an independent predictor of treatment failure, suggesting that very short treatment courses may be insufficient. These apparently conflicting findings become reconcilable once two distinct variables are separated: the duration of rifampicin itself, and the duration of the total antibiotic course. Scheper et al. evaluated a deliberately short rifampicin course embedded within an otherwise standard total treatment duration, whereas in the Becker et al. cohort a short rifampicin course more likely reflected premature treatment discontinuation, early clinical failure prompting rifampicin cessation, or a shorter total antibiotic course overall—a form of confounding by indication rather than a true causal effect of rifampicin duration per se. Overall, the optimal duration of rifampicin therapy cannot be firmly determined from the available evidence: most observational cohorts and meta-analyses included patients treated with rifampicin for approximately 3 months, and the minimum effective duration remains to be established in adequately powered randomised controlled trials, ideally designed to separate rifampicin-specific duration from total antibiotic course duration.

### 3.7. Alternatives to Rifampicin

When rifampicin is contraindicated, poorly tolerated, or ineffective, several other antibiotics have documented anti-biofilm activity and may serve as companions or substitutes; a broader treatment of the wider anti-biofilm antibiotic landscape beyond rifampicin, including fluoroquinolones, fosfomycin, tetracyclines, fusidic acid, glycopeptides, lipopeptides, lipoglycopeptides, and colistin, is available in a recent complementary review [91].

#### 3.7.1. Rifabutin

Rifabutin is a rifamycin derivative with anti-biofilm activity comparable to rifampicin but with significantly less CYP450 induction, substantially reducing DDI potential. Drug interactions caused by rifampicin are approximately twice as potent as those caused by rifabutin at standard doses (300 mg/day), making rifabutin clinically preferable in patients receiving antiretrovirals, DOACs, immunosuppressants, or other CYP3A4/CYP2C9 substrates.

Monk et al. [92] reported encouraging results in seven patients receiving rifabutin instead of rifampicin for staphylococcal implant infections. Leal et al. [61] evaluated 87 staphylococcal PJI patients: rifabutin was successfully substituted in eligible patients with only 3.4% having a contraindication versus 18.4% to rifampicin. In vitro antibacterial and anti-biofilm activity of rifabutin against staphylococci is comparable to rifampicin [93]; moreover, cross-resistance is not universal—certain rpoB mutants retain rifabutin susceptibility, making it occasionally useful even in rifampicin-resistant isolates.

#### 3.7.2. Emerging Alternatives

Delafloxacin, a new-generation fluoroquinolone with enhanced anti-staphylococcal and intracellular activity, and dalbavancin, a long-acting lipoglycopeptide enabling once-weekly or single-dose administration, represent promising agents for difficult-to-treat OAI. Both have been proposed as potential companion agents for rifampicin rather than outright replacements: in vitro, dalbavancin combined with rifampicin shows additive activity against staphylococcal biofilm [94], and rifampicin combined with delafloxacin significantly reduces biofilm biomass, including in the acidic microenvironment typical of infected bone and joint tissue [95]. These combinations could allow once-weekly dosing or improved tissue penetration while preserving rifampicin’s anti-biofilm contribution, but clinical data remain limited to in vitro and small case-series experience, and comparative trials against standard rifampicin–fluoroquinolone regimens are still needed.

Bacteriophage therapy combined with antibiotics is under active investigation for polymicrobial or multidrug-resistant FRI and PJI cases refractory to conventional treatment. In vitro, combinations of lytic phages with vancomycin or rifampicin produce synergistic bactericidal activity against staphylococcal biofilm, with phages disrupting the biofilm matrix and re-sensitising embedded bacteria to antibiotics, including rifampicin [96]. Should anti-staphylococcal phage–antibiotic combinations mature into validated clinical regimens, they could in principle reshape rather than simply supplement current rifampicin combination indications: by directly disrupting biofilm architecture, an effective phage partner might reduce reliance on rifampicin for anti-biofilm activity specifically, allowing rifampicin to be reserved for a narrower set of resistant or refractory cases, or conversely could be combined with rifampicin from the outset to reduce the inoculum against which resistant mutants are selected; this remains speculative until comparative clinical data are available. A systematic review of 51 patients treated with phage therapy for bone and joint infection—predominantly by topical administration and combined with antibiotics in 79% of cases—reported an overall success rate of 71%, but no randomised controlled trial has yet been performed, administration protocols remain highly heterogeneous, and phage–antibiotic matching requires specialised laboratory support [97]. Phage therapy for OAI therefore remains investigational, currently restricted to compassionate-use protocols in reference centres, and should be regarded as a salvage strategy used alongside—rather than in place of—rifampicin-based antibiotic therapy when a rifampicin-susceptible staphylococcal component is present.

#### 3.7.3. Novel Local Delivery Modalities: Intra-Articular and Continuous Antibiotic Perfusion

Continuous local antibiotic perfusion, delivered via indwelling intra-articular or intramedullary catheters combined with negative-pressure drainage, has emerged as a technique to achieve sustained, high local antibiotic concentrations while limiting systemic toxicity. Choe et al. [98] described the principles, technical aspects, and reported clinical outcomes of continuous local antibiotic perfusion for orthopaedic infections, including FRI and PJI. An ongoing multicentre randomised controlled trial (Ji et al. [99]) is comparing intra-articular antibiotic infusion with standard intravenous antibiotic administration for infected knee arthroplasty, and its results should help define the place of this technique relative to systemic therapy. Where rifampicin cannot be used systemically because of intolerance, drug–drug interactions, or resistance, local perfusion of a companion agent such as vancomycin may partly recreate the high-concentration, biofilm-targeted exposure that rifampicin otherwise provides orally; conversely, when rifampicin is used systemically, local perfusion of a partner antibiotic could in principle allow dose reduction in that partner agent. These techniques remain investigational for FRI and PJI, and comparative data against standard systemic rifampicin-based regimens are still lacking. Unlike gentamicin, no dedicated commercial local or intraosseous rifampicin delivery system (e.g., loaded beads or a resorbable carrier) is available for routine use: admixing rifampicin into polymethylmethacrylate bone cement interferes with cement polymerisation and degrades its mechanical properties, which has confined rifampicin to the systemic oral route in current practice, while local antibiotic carriers for osteoarticular infection continue to rely on gentamicin, tobramycin, or vancomycin [100].

#### 3.7.4. Biofilm-Degrading Enzymes

Biofilm-degrading (lytic) enzymes—including DNases, dispersin B, and other glycoside hydrolases—target the extracellular polymeric matrix that shields bacteria from antibiotics and host defences, offering a mechanistically distinct complement to rifampicin’s direct anti-biofilm bactericidal activity. In a rat implant-associated infection model, Buzisa Mbuku et al. [34] showed that a hydrogel combining biofilm-degrading enzymes with antibiotics reduced bacterial burden to undetectable levels; this effect was substantially attenuated when systemic rifampicin was omitted from the regimen, indicating that enzymatic biofilm disruption potentiates rather than replaces rifampicin’s activity. In that model, local vancomycin delivery also appeared to protect against emergence of rifampicin resistance, suggesting a complementary strategy in which enzymatic biofilm dispersal exposes embedded bacteria to systemic rifampicin, while local companion-antibiotic delivery limits the selective pressure for rifampicin-resistant mutants. These findings, while preliminary and derived from a preclinical model, position lytic enzymes as a promising adjunct to—rather than an alternative to—rifampicin-based therapy in OAI.

### 3.8. Evidence Gaps and Future Directions

#### 3.8.1. Limitations of Current Evidence

Despite nearly three decades of clinical use, the evidence base for rifampicin in OAI suffers from major limitations: very few randomised trials (only three RCTs for PJI, all small with significant methodological limitations); predominance of retrospective observational studies with heterogeneous populations, surgical strategies, failure definitions, and rifampicin dosing regimens; insufficient evidence for non-staphylococcal pathogens (*streptococci*, *Cutibacterium*, *enterococci*); absence of robust pharmacodynamic data (AUC/MIC ratio targets) in OAI; inadequate data on optimal timing, minimum treatment duration, and predictors of treatment response; and under-representation of FRI and osteomyelitis in large cohort studies and meta-analyses.

These limitations are compounded by additional methodological caveats. The effect measures reported across studies (relative risk, odds ratio, hazard ratio) reflect the statistical choices of the original investigators and are not directly interchangeable; comparisons across the studies and meta-analyses summarised in Supplementary Tables S2, S3, and S5 should therefore be interpreted qualitatively rather than as directly pooled estimates. None of the observational cohorts or meta-analyses discussed in this review underwent a formal risk-of-bias assessment (e.g., Cochrane RoB2 for randomised trials, Newcastle–Ottawa Scale for observational studies) or GRADE-based certainty rating; such an assessment would help calibrate the strength of the practical recommendations offered here, particularly those—such as the delay before introduction and the minimum treatment duration discussed in Section 3.6—that currently rest on single studies with conflicting counter-evidence. Finally, data on rifampicin use in pregnancy, paediatric osteoarticular infection, and in patients with significant renal or hepatic impairment are essentially absent from the literature reviewed here and could not be addressed. Illustrating this heterogeneity, the Kruse et al. meta-analysis [46] is cited at several points in this review with distinct pathogen- and procedure-specific subgroup estimates—including for *streptococci* and fracture-related infection—that are not directly comparable with one another or with its pooled all-pathogen estimate.

#### 3.8.2. Ongoing Clinical Trials

The RiCOTTA trial [23] is the most significant ongoing study. It will assess whether antibiotic monotherapy (without rifampicin) is non-inferior to rifampicin combination during the oral phase of staphylococcal PJI managed by DAIR. If non-inferiority is confirmed, a more individualised approach—reserving rifampicin for high-risk patients or mature biofilm situations—would become feasible.

The RIFACute trial [73] will specifically address the impact of rifampicin in *C. acnes* PJI.

#### 3.8.3. Priority Research Areas

Priority areas include: well-powered RCTs for *streptococcal*, *Cutibacterium*, and *enterococcal* implant infections; biomarkers predictive of rifampicin response; tissue-level pharmacokinetic/pharmacodynamic studies within infected bone and joint compartments; local rifampicin delivery systems compatible with orthopaedic mechanical constraints; combined rifampicin + bacteriophage or rifampicin + anti-biofilm molecule approaches for refractory infections; and European and international epidemiological surveillance of rifampicin resistance in OAI isolates.

## 4. Conclusions

Rifampicin remains a valuable and largely irreplaceable therapeutic tool in the management of Gram-positive osteoarticular infections, particularly staphylococcal OAI with implant retention. Its unique bactericidal activity against stationary-phase bacteria, biofilm-penetrating capacity, and excellent oral bioavailability place it in a distinct category among available antibiotics.

The optimal conditions for rifampicin use are now better defined: mandatory combination therapy (never monotherapy) and fluoroquinolone as preferred partner. Observational data further suggest that introduction after surgical source control (delay >5 days post-debridement associated with lower failure in a single multicentre cohort [38]) and adequate duration (≥14 days associated with better outcomes in one retrospective series [40], although a short 5-day course performed comparably to prolonged combination therapy in another cohort [90]) may improve outcomes; however, neither threshold has been validated in a randomised trial, and both should be regarded as pragmatic, evidence-informed defaults rather than firm rules. The benefit is greatest for DAIR procedures and one-stage revision; evidence is weaker and less consistent for two-stage procedures. Although much of the underlying evidence discussed in this review derives from PJI cohorts specifically, the pathophysiological rationale for rifampicin—biofilm on a retained implant surface—applies equally to FRI and to implant-associated osteomyelitis with hardware in place; the recommendations above should therefore be read as applying broadly to implant-related infections with retained hardware, not to PJI alone, while acknowledging that FRI- and osteomyelitis-specific outcome data remain comparatively sparse (Section 3.3.2 and Section 3.3.3, Supplementary Table S3).

Pathogen-specific data remain heterogeneous beyond *staphylococci*. For streptococci and *Cutibacterium*, a favourable trend exists without strong recommendation. For *enterococci*, clinical evidence is essentially absent. Dedicated randomised trials for these pathogens are urgently needed.

Resistance emergence, tolerability issues and clinically significant DDI remain the principal limitation, necessitating judicious use and development of alternatives—rifabutin, delafloxacin, dalbavancin, bacteriophages—for situations where rifampicin is contraindicated, poorly tolerated, or ineffective. Multidisciplinary management in dedicated bone and joint infection referral centres remains the most consistently identified prognostic determinant across all published studies, independent of antibiotic choice [101].

## Data Availability

No new data were created or analyzed in this study. Data sharing is not applicable to this article.

## Supplementary Materials

The following supporting information can be downloaded at: https://www.mdpi.com/article/10.3390/antibiotics15090872/s1, Table S1: In Vitro Anti-Biofilm Activity of Rifampicin in Osteoarticular Infections; Table S2: Comprehensive Summary of Studies and Meta-Analyses on Rifampicin-Based Combinations in Prosthetic Joint Infections (All Surgical Strategies); Table S3: Summary of Studies on Rifampicin in Fracture-Related Infections; Table S4: Rifampicin Efficacy by Pathogen in Implant-Associated Osteoarticular Infections: Summary of Evidence and Recommendations; Table S5: Summary of Studies on Rifampicin in *Streptococcal* Prosthetic Joint Infections; Table S6: Antibiotic Combinations with Rifampicin: Dosing, Pharmacokinetic Interactions, and Clinical Evidence in Osteoarticular Infections.
