# Supplementary material for: Rifampicin in Osteoarticular Infections: A Comprehensive Review of Current Evidence
Source: Antibiotics (Basel). 2026 Sep 7;15(9):872. doi: 10.3390/antibiotics15090872 (PMC13603077; doi:10.3390/antibiotics15090872)
Supplement: Supplementary file 1 [file antibiotics-15-00872-s001.zip › antibiotics-4523168-supplementary.pdf]

Supplementary Materials

Table S1. In Vitro Anti-Biofilm Activity of Rifampicin in Osteoarticular Infections

Study (Year)	Pathogen / Model	Antibiotics Tested	Key Findings
Molina-Manso et al. (2013) [10]	*S. aureus*, *S. epidermidis* (PJI, n=32)	9 agents including rifampicin, tigecycline	Rifampicin + tigecycline: lowest MBECs; no single agent achieved complete eradication
Tang et al. (2013) [20]	MRSA biofilm (static model)	Rifampicin ± linezolid, fosfomycin, minocycline, fusidic acid, tigecycline	≥100-fold reduction in bacterial burden with all 5 partner antibiotics; fewer resistant mutants vs monotherapy
Gidari et al. (2020) [33]	MSSA, MRSA (mature 24 h biofilm)	Tedizolid + rifampicin	Biofilm disaggregation; bactericidal at clinical concentrations; complete prevention of rifampicin resistance in vitro
Alagboso et al. (2022) [21]	*S. aureus* intracellular (osteoblasts)	Rifampicin 8 μg/mL	Complete eradication of intracellular *S. aureus*; restoration of extracellular matrix mineralisation at 21 days
Liu et al. (2025) [13]	*S. aureus* agr-mutant biofilm	Rifampicin vs comparators	Quorum sensing-controlled efflux exporter mechanism explains uniquely superior activity against metabolically quiescent biofilm
Furustrand Tafin et al. (2012) [69]	*Cutibacterium acnes* biofilm	Rifampicin combinations vs monotherapy	Rifampicin combinations superior to monotherapy against *C. acnes* biofilm; low MIC values confirmed in vitro
Holmberg et al. (2015) [74]	*E. faecalis* PJI isolates (biofilm)	Ciprofloxacin + rifampicin → linezolid + rifampicin sequence	Most effective against *E. faecalis* biofilm; sequential combination retained activity; less resistance emergence than monotherapy

Table S2. Comprehensive Summary of Studies and Meta-Analyses on Rifampicin-Based Combinations in Prosthetic Joint Infections (All Surgical Strategies)

Study [Ref] Year	Design	N	Surgical Strategy	Pathogen	RIF Dose	Companion Drug	Duration	Success +RIF	Success −RIF	Effect Size	95% CI	P value
Zimmerli et al. [11] 1998	RCT, DB, multicentre	69	DAIR (implant retention)	*S. aureus*, CoNS (staphylococci)	300 mg BD or 450	Ciprofloxacin 750 mg BD	3–6 mo (hip/knee)	100%	58%	Absolute diff +42%	NR	<0.001

Study [Ref] Year	Design	N	Surgical Strategy	Pathogen	RIF Dose	Companion Drug	Duration	Success +RIF	Success −RIF	Effect Size	95% CI	P value
					mg BD							
Karlsen et al. [36] 2020	RCT, open-label, single-centre	99	DAIR	Staphylococci (MSSA/ MRSA/CoNS)	600 mg OD	Cloxacillin (oral)	3–6 mo	74%	72%	RR 1.03	0.73 – 1.45	NS
Lora-Tamayo et al. [37] 2016	RCT, open-label, multicentre	63	DAIR	*S. aureus*, CoNS (staphylococci)	300 mg BD	Levofloxacin 500 mg OD–BD	8 wk vs 3–6 mo	91.7%	95.0%	Diff +3.3% (non-inferiority)	−11.7 to +18.3%	NS (NI)
Aboltins et al. [41] 2007	Prospective cohort, single-centre	20	DAIR (implant retention)	Staphylococci (MSSA/CoNS)	300 mg BD	Fusidic acid 500 mg TID	Median 32 mo f/u	90% infection- free at f/u	Historical comparator	—	—	—
Byren et al. [42] 2009	Retrospective cohort, single-centre	112	DAIR	Staphylococci (mixed)	Variable	Variable (FQ-based)	3 mo (majority)	82% (recurrence-free)	No comparator arm	—	—	—
Lora-Tamayo et al. (REIPI) [43] 2013	Retrospective multicentre cohort (Spain, 11 centres)	345 (81 MRSA, 264 MSSA)	DAIR	*S. aureus* (MSSA/MRSA)	Variable	Variable (FQ majority)	Median 90 days	55% overall; RIF benefit in homogenising MSSA vs MRSA	Comparison within cohort	OR NR	NR	NR
Beldman et al.	Multicentre retrospective	669	DAIR (staphylococcal)	*S. aureus*, CoNS (MSSA/MRSA)	300 mg BD or	FQ (levo/cipro) or	Variable (medi	67.8% (1 yr success)	45.8%	ARR 22%	NR	<0.001

Study [Ref] Year	Design	N	Surgical Strategy	Pathogen	RIF Dose	Companion Drug	Duration	Success +RIF	Success −RIF	Effect Size	95% CI	P value
[38] 2021	cohort (4 countries, 6 centres, 1997–2017)				450/600 mg OD	clindamycin	an 12 wk)					
Tornero et al. [44] 2016	Retrospective multicentre cohort (ESGIAI, multinational)	143	DAIR	Staphylococci	300 mg BD or 600 mg OD	FQ vs linezolid/ clinda/ TMP-SMX	Variable	RIF+FQ: 91.7%; RIF+LZD: 72.2%; RIF+TMP-SMX: 62%	Intra-cohort	OR NR	NR	FQ vs others: P = 0.003
Kramer et al. (ESGIAI) [47] 2023	Multicentre observational cohort (ESGIAI, multinational)	375	One-stage and two-stage revision	Staphylococci (*S. aureus*/CoNS)	Variable	Variable	Variable	No overall benefit; subgroup 2-stage: favours RIF	No significant overall benefit	HR NR	NR	NS (overall)
Lora-Tamayo et al. [50] 2021	Retrospective multicentre cohort (Spain, 7 centres)	215	Predominantly two-stage revision	*S. aureus* (MSSA/MRSA)	Variable; RIF ≥21 days in 55.4 %	Variable	Variable	RIF not independently predictive in multivariate analysis	Comparator: no RIF	OR NR	NR	NS
Zhang et al. [17] 2025	Multicentre retrospective cohort	227	DAIR + explantation (mixed)	Staphylococci	Variable	Variable	Variable	79.75%	73.65%	—	—	0.083 (NS)

Study [Ref] Year	Design	N	Surgical Strategy	Pathogen	RIF Dose	Companion Drug	Duration	Success +RIF	Success −RIF	Effect Size	95% CI	P value
	(China, 2014–2023)											
Nitta et al. [58] 2024	Claims database study 52,588 hip arthroplasty patients	NR (large cohort)	Implant retention (DAIR)	Staphylococci (MRSA/MSSA)	Variable	Variable (RIF-combination)	Variable	Significantly reduced revision surgery risk	No-RIF arm	OR 0.61	0.47–0.80	<0.001
Leal et al. [61] 2025	Retrospective cohort, single-centre	87	DAIR (IDSA criteria for RIF)	Staphylococci (MSSA/MRSA/CoNS)	300 mg BD or 600 mg OD	Variable (FQ-based)	Variable	71.4% (full RIF course completers)	DDI group: lower success	Sig. association	NR	<0.05
Scheper et al. [39] 2021	Systematic review & meta-analysis (62 studies)	NR	DAIR (hip & knee)	Staphylococci	Variable	Variable	Variable	Pooled RR for success 1.10	Without RIF	RR 1.10	1.00–1.22	0.04
Kobayashi et al. [45] 2024	Systematic review & meta-analysis (27 studies)	NR	All strategies (DAIR, 1-stage, 2-stage)	Staph, CoNS, Cutibacterium, Streptococcus	Variable	Variable	Variable	RR benefit maintained in DAIR & 1-stage	Without RIF	RR 1.14	1.05–1.24	<0.001
Kruse et al. [46] 2022	Systematic review & meta-analysis	NR	DAIR + revision	Mixed (Staph predominant)	Variable	Variable	Variable	OR for failure 0.57 (favours RIF)	Without RIF	OR 0.57	0.41–0.78	<0.001

Study [Ref] Year	Design	N	Surgical Strategy	Pathogen	RIF Dose	Companion Drug	Duration	Success +RIF	Success −RIF	Effect Size	95% CI	P value
	(22 comparative studies)											
Yusuf et al. [48] 2024	Systematic review & meta-analysis (18 studies)	NR	DAIR / two-stage	Staph/CoNS/ Streptococci	Variable	Variable	Variable	Benefit in DAIR; no significant benefit in 2-stage (~90% both)	Without RIF	RR NR	NR	DAIR: P<0.05; 2-stage: NS
Becker et al. [40] 2020	Retrospective cohort, single-centre	113	DAIR	Staphylococci (early-onset)	300 mg BD or 600 mg OD	FQ (majority)	Short (<14 d) vs long (>14 d)	Duration >14 d: significantly better outcome	RIF <14 days	HZ NR	NR	0.0017
Krizsan et al. [59] 2022	Retrospective cohort, single-centre	80	Two-stage revision	Staphylococci	Variable	Variable	Variable	RIF-susceptible: 92.5% cure	RIF-resistant: 60.0% cure	Diff 32.5%	NR	<0.05
Aunon et al. [60] 2026	Retrospective cohort, 15 yr, single-centre	NR	Mixed	*S. epidermidis* (CoNS)	Variable	Variable	Variable	MDR/XDR & RIF resistance: indep. failure predictors	RIF-susceptible comparator	OR 8.01 (double resist.)	2.82 – 22.77	<0.001

Table S3. Summary of Studies on Rifampicin in Fracture-Related Infections

Study (Year)	Design / N	Outcome	Key Comment
Kobayashi et al. 2024 [45]	Meta-analysis; spinal-instrumentation-infection subgroup within a 27-study dataset	Benefit of rifampicin-based regimens in DAIR-equivalent procedures on osteosynthesis hardware	Extrapolated from a predominantly PJI-focused meta-analysis, not an FRI-specific cohort
Kruse et al. 2022 [46]	Meta-analysis (22 comparative studies); non-DAIR-exclusive surgical strategies	Protective effect of rifampicin-containing regimens (OR 0.47; 95% CI 0.27–0.80)	FRI not isolated as a distinct subgroup; pooled across mixed surgical strategies
McNally et al. [55]	Cohort; local antibiotic carrier for FRI	FRI recurrence reduced from 18.7% to 10.0% (HR 0.48; 95% CI 0.29–0.81)	Effect attributed to local antibiotic implantation, not systemic rifampicin specifically
Buijs et al. 2022 [56]	Largest reported FRI-DAIR cohort; rifampicin given to 65% of patients	Rifampicin use not an independent predictor of treatment success on multivariable analysis	First dedicated FRI-DAIR cohort testing rifampicin independently; PJI benefit not replicated
Corrigan et al., EAT-FRI, 2023 [57]	Multicentre cohort	Guideline non-compliance, including rifampicin misuse/non-use, not associated with worse outcomes (12.1% vs 13.2% failure, P=0.87)	Suggests rifampicin's marginal effect size in FRI may be smaller than in PJI

Table S4. Rifampicin Efficacy by Pathogen in Implant-Associated Osteoarticular Infections: Summary of Evidence and Recommendations

Pathogen	Setting & Level of Evidence	Key Clinical Outcomes	Preferred Combination	Key Notes / Cautions
S. aureus (MSSA/MRSA)	PJI (DAIR) Strong: 3 RCTs + multiple	Failure 32.2% vs 54.2% without RIF (Beldman 2021 [38]; n=669). RR benefit 1.27 (1.11–1.45) (Kobayashi 2024 [45]). Pooled RR success 1.10	Levofloxacin (preferred) or ciprofloxacin + RIF 300 mg BD or 600 mg OD	MSSA and MRSA: comparable outcomes if companion drug matched. Dual levofloxacin + RIF resistance: OR 8.01 (2.82–

Pathogen	Setting & Level of Evidence	Key Clinical Outcomes	Preferred Combination	Key Notes / Cautions
	meta-analyses	(1.00–1.22) (Scheper 2021 [39]). Benefit in 1-stage: RR 1.11 (1.03–1.20); weaker in 2-stage.		22.77) for failure (Aunon 2026 [60]).
CoNS	PJI (DAIR) Moderate: observational data	Scheper 2021 [39]: success hip 83%, knee 73% — superior to *S. aureus*. Positive association with RIF use across cohorts.	Fluoroquinolone (levofloxacin) + RIF 300 mg BD or 600 mg OD	Primary RIF resistance: 6.8–20% (Edslev 2025 [63]; Lourtet-Hascoet 2018 [62]). Test susceptibility before initiating. 11 rpoB mutations identified, several novel.
Streptococci (agalactiae, dysgalactiae, viridans)	PJI (DAIR) Conflicting evidence	Kobayashi 2024 [45]: RR 1.18 (1.02–1.38), P=0.03. Kruse 2022 [46]: OR failure 0.36 (0.14–0.93). Yusuf 2024 [48] & Gerritsen 2023 [68]: no significant benefit after adjustment. Gerritsen MA (n=1,367 pts): pooled DAIR success 71%; knee > hip prognosis.	Levofloxacin + RIF or beta-lactam (based on susceptibility)	Evidence insufficient for strong recommendation. No consensus across meta-analyses. Penicillins remain backbone; role of RIF adjunctive and uncertain.
Cutibacterium acnes	PJI (shoulder/hip) Inconsistent evidence	Kobayashi 2024 [45]: RR 1.11 (1.01–1.22), significant. Kusejko 2021 [71] (n=187): no benefit after adjustment. High baseline cure rates (>80%) limit statistical power.	Amoxicillin or levofloxacin + RIF 300 mg BD or 600 mg OD	High baseline cure rates limit power to show benefit. Diagnosis often delayed (indolent course). RIFACute trial [73] ongoing (completion 2027).
Enterococci (*E. faecalis*/*E. faecium*)	PJI Very limited (no RCT)	In vitro (Holmberg 2015 [74]): ciprofloxacin + RIF → linezolid + RIF most effective vs *E. faecalis* biofilm. Small observational series suggest lower failure in early infections (<30 d).	Ampicillin (*E. faecalis*) or linezolid (*E. faecium*) + RIF	No randomised trial. Clinical evidence essentially absent. RIF only in early implant infections. *E. faecium*: intrinsic ampicillin resistance; use linezolid or daptomycin.

Table S5. Summary of Studies on Rifampicin in Streptococcal Prosthetic Joint Infections

Study (Year)	Design / N	Outcome	Key Comment
Fiaux et al. 2016 [65]	Retrospective multicentre cohort; n=95; 4 French centres	70.5% overall remission. Univariate: trend favouring RIF + levofloxacin. Multivariate: only prosthesis location (hip vs knee) independently prognostic.	No independent RIF benefit in multivariate analysis; confounded by small sample and centre heterogeneity
Lora-Tamayo et al. 2017 [66]	Multicentre cohort; n=462; 52% haematogenous; 34.4% *S. agalactiae*	42.1% failure at median 62 days. RIF early use: protective (HR 0.98/treatment-day in first 30 days). Beta-lactam >21 days: independently protective.	RIF not independently significant in multivariate. Surgical and host factors dominated.
Gerritsen et al. 2023 [68]	Systematic review/meta-analysis; 25 studies; n=1,367 streptococcal DAIR	Pooled DAIR success 71% (95% CI 64–77%). Knee 76% vs hip 58%. RIF group: 84% vs no-RIF 74%; unadjusted difference only.	No clear benefit after meta-analysis adjustment. Knee > hip prognosis confirmed.
Borens et al. 2025 [67]	Retrospective cohort, single-centre; n=62 streptococcal PJI	23% failure at 2 years — comparable to non-streptococcal PJI (21%; P=NS). *S. dysgalactiae* and DAIR independently associated with failure.	RIF not independently protective in multivariate analysis.
Saltiel et al. 2022 [72]	Systematic review	No significant benefit (OR 1.84; 95% CI 0.90–3.76)	Trend favouring RIF without statistical significance; Bayesian model suggested against routine use
Kruse et al. 2022 [46]	Meta-analysis (22 comparative studies)	Significant protective effect for streptococcal PJI (OR 0.36; 95% CI 0.14–0.93; P=0.03)	Heterogeneous populations; limited streptococcal-specific data
Kobayashi et al. 2024 [45]	Meta-analysis (27 studies)	Significant benefit (RR 1.18; 95% CI 1.02–1.38; P=0.03), especially with fluoroquinolone	Moderate heterogeneity; effect size smaller than for staphylococci

Table S6. Antibiotic Combinations with Rifampicin: Dosing, Pharmacokinetic Interactions, and Clinical Evidence in Osteoarticular Infections

Combination	RIF Dose	Companion Dose	PK Interaction	Clinical Efficacy	Recommendation
RIF + Levofloxacin	300 mg BD or 600 mg OD (fasting)	Levofloxacin 500 mg OD–BD	FQ AUC reduced 30–50%; clinical benefit preserved at standard doses	Failure 14% — best outcome (Beldman 2021 [38]). RR benefit maintained (Kobayashi 2024 [45]). Establish stable FQ dosing before introducing RIF.	FIRST-LINE (Preferred)
RIF + Ciprofloxacin	300 mg BD or 600 mg OD (fasting)	Ciprofloxacin 750 mg BD	CIP AUC reduced; higher staphylococcal MIC vs levofloxacin	100% cure MSSA/CoNS (Zimmerli 1998 [11]). Historically most used; levofloxacin preferred.	FIRST-LINE
RIF + Moxifloxacin	300 mg BD or 600 mg OD (fasting)	Moxifloxacin 400 mg OD	AUC reduced 67–85% in target tissue (Slater 2022 [76])	No significant difference vs RIF/levofloxacin (Wouthuyzen-Bakker 2018 [77], n=58). Tissue concentrations likely remain above MIC breakpoints.	Alternative — Caution: QT prolongation ; DDI
RIF + Clindamycin	300 mg BD or 600 mg OD (fasting)	Clindamycin 300–450 mg TID or QID	Reduced [clindamycin]; similar clinical outcomes despite lower serum levels	Failure 14% (Beldman 2021 [38]). Cure rate 94% (Leijtens 2019 [80]). Anti-biofilm/anti-adhesion properties independent of RIF.	VALID if FQ contraindicated or resistant
RIF + Cotrimoxazole	300 mg BD or 600 mg OD (fasting)	TMP 160 mg / SMX 800 mg BD	Reduced [TMP-SMX] via CYP450 induction; clinically significant	Failure 38% (Beldman 2021 [38]) — worst outcome of all combinations tested.	DISCOURAGED — avoid as primary companion; reserve only if no other combination is feasible
RIF + Linezolid	300 mg BD or 600 mg OD (fasting)	Linezolid 600 mg BD	Reduced [linezolid] via CYP450 induction	Trend toward more failures (Kobayashi 2024 [45]). Only combination independently associated with higher failure (Tornero 2016 [44]). Utility in MRSA/XDR only.	AVOID unless no alternative (MRSA, XDR)
RIF + Fusidic acid	300 mg BD or 600 mg OD (fasting)	Fusidic acid 500 mg TID	Mutual pharmacokinetic interactions; both agents mutually reduced	Cure ~55% vs 50% with RIF/ofloxacin (Drancourt 1997 [86]). Phase 2 RCT terminated due to interactions.	LAST RESORT — when alternatives exhausted

Combination	RIF Dose	Companion Dose	PK Interaction	Clinical Efficacy	Recommendation
RIF + Doxycycline	300 mg BD or 600 mg OD (fasting)	Doxycycline 100 mg BD	Reduced [doxycycline] via CYP450 induction	Very limited clinical OAI data. Good oral bioavailability; broad Gram-positive spectrum. Mainly when eradication not achievable.	Suppressive therapy only — very limited evidence
RIF + Delafloxacin	300 mg BD or 600 mg OD (fasting)	Delafloxacin 450 mg BD	PK interaction not formally characterised; in vitro combination retains anti-biofilm activity	Significant reduction in biofilm biomass in vitro, including in acidic microenvironments (de Andrade 2026 [95]). No clinical efficacy data yet.	INVESTIGATIONAL — in vitro evidence only
RIF + Dalbavancin	300 mg BD or 600 mg OD (fasting)	Dalbavancin 1,500 mg single dose or 500 mg weekly	No clinically significant CYP-mediated interaction (dalbavancin is not a CYP450 substrate)	Additive anti-biofilm activity against *S. aureus* in vitro (Jacob 2021 [94]). Once-weekly dosing may allow outpatient regimens. No clinical efficacy data yet.	INVESTIGATIONAL — in vitro evidence only
RIF + Bacteriophages	300 mg BD or 600 mg OD (fasting)	Lytic phage cocktail (protocol-dependent; compassionate use)	No pharmacokinetic interaction; phages disrupt biofilm matrix and re-sensitise embedded bacteria to rifampicin	Synergistic bactericidal activity in vitro (Kolenda 2020 [96]); 71% overall clinical success in a 51 patient systematic review, mostly combined with antibiotics (Genevière 2021 [97]). No RCT.	SALVAGE — compassionate-use / reference centres only
